# Depression, Ulcers and Confusion – A Clinical Case of Behçet’s Disease with Psychiatric Presentation

**DOI:** 10.1192/j.eurpsy.2024.990

**Published:** 2024-08-27

**Authors:** F. M. H. Agostinho, M. B. Resende, M. J. Amaral, R. Nogueira, V. P. Falcão, D. Cotovio

**Affiliations:** Psychiatry, Hospital Beatriz Angelo, Lisbon, Portugal

## Abstract

**Introduction:**

Behçet’s disease, a rare autoimmune disorder, can present a challenging diagnostic puzzle, particularly when neuropsychiatric symptoms take the forefront. In this case study, we delve into the diagnostic process of a 43-year-old patient without prior psychiatric history, who initially presented with depressive and catatonic symptoms. The trajectory from psychiatric admission to a final diagnosis of Behçet’s disease with neuropsychiatric involvement underscores the importance of interdisciplinary collaboration and the consideration of rare diseases in psychiatric assessment. Clinical remission was achieved with immunosuppressive therapy.

**Objectives:**

Presentation of a clinical case of Behçet’s disease with neuropsychiatric manifestations.

**Methods:**

Review of the patient’s clinical data in SOARIAN platform and research on UptoDate and Pubmed using the terms “Catatonia,” “Behçet disease,” “Neuro-Behçet,” and “Psychiatry.”

**Results:**

We present a clinical case of a 43-year-old patient, originally from India, not fluent in Portuguese or English, with no prior psychiatric history, who presented to the emergency department exhibiting mutism and was admitted to the psychiatry department with the diagnostic hypothesis of depressive episode with psychotic and catatonic symptoms. During hospitalization, severe vitamin deficiencies, gastrointestinal symptoms (vomiting, abdominal pain, and hematochezia), and gynecological symptoms (dyspareunia and vaginal discharge) were observed. From a psychiatric perspective, in addition to depressive and psychotic symptoms, atypical symptomatology incongruent with the initial diagnosis was identified, raising suspicion of an “organic” disease. There was an atypical fluctuation in symptoms, with periods of severe behavioral disorganization interspersed with periods of apathy and psychomotor retardation, significant alterations in attention and memory, and executive deficits. Additionally, there was a poor response to psychiatric medication and electroconvulsive therapy. A colonoscopy revealed ulcers at the ileocecal valve, and gynecological lesions suggestive of a vasculitic process were observed. Autoimmunity testing showed positivity for HLA B51/52. Given the neuropsychiatric, gastrointestinal, and gynecological manifestations, along with suggestive autoimmunity, the diagnosis of Behçet’s Disease with neurological involvement was established. Clinical remission was achieved only with immunosuppressive therapy. The case is enriched by the complex diagnostic journey, multiple complications encountered (including valproic acid-induced encephalopathy), and the challenges faced in treating neuropsychiatric manifestations.

**Conclusions:**

This clinical case exemplifies the challenges in diagnosing a systemic disease with primary psychiatric presentation, as well as the therapeutic success resulting from multidisciplinary collaboration in a public hospital.

**Disclosure of Interest:**

None Declared

